# Data on Swiss grape growers’ production, pest and labour management decisions

**DOI:** 10.1016/j.dib.2025.112421

**Published:** 2025-12-24

**Authors:** Philipp Höper, Lucca Zachmann, Robert Finger

**Affiliations:** Agricultural Economics and Policy Group, ETH Zurich, Switzerland

**Keywords:** Viticulture, Pesticide use, Sustainable agriculture, Pest management, Adoption analysis, Agricultural labour

## Abstract

This dataset comprises survey responses from 489 grape growers in Switzerland, focusing on their decisions related to production, pest management, risk management, behavioural factors, and labour management. The online survey was conducted in early spring 2025 and includes details on grape variety selection, farm practices, and characteristics of both the farmers and their farms. Additional data covers all other relevant pest management strategies targeting weeds, insects, and fungal threats. Farmer-specific attributes such as education, gender, age, and sources of information were recorded, alongside general labour force characteristics and perceptions regarding recruitment, and mechanization. Behavioural factors including risk and time preferences, self-efficacy, and locus of control were assessed using self-report scales. Farm-level data includes marketing approaches, labels and production systems, agri-environmental programs, and pesticide application equipment. The survey responses were linked with environmental variables—such as temperature and rainfall—and spatial data on the infection risk of *Oidium* and *Peronospora viticola*. Innovatively, the data contains the adoption stage of growers (not just binary adoption) for four key pesticide-reducing practices (i.e. the plantation of fungus-resistant varieties, the use of plant resistance inducers, inorganic materials and mechanical weeding), along with their views on influencing factors and labour demands associated with these measures. This dataset provides an extensive resource for standalone analyses on production, pest management, risk management, behavioural factors, and labour management, as well as for use in meta-analyses or as part of a panel dataset combined with previous similar surveys.

Specifications Table

**Table:** 

Subject	Social Sciences
Specific subject area	Socioeconomic impacts of pest management practices in Swiss Viticulture, including production and labour management decisions.
Type of data	CSV file, Raw, Partly filtered (for confidentiality reasons)
Data collection	The online questionnaire was distributed in German, French and Italian via LimeSurvey to a sample of 4’136 grape growers in Switzerland from February 28th to April 6th, 2025. The population of Swiss vineyards totals just over 4000 (including both commercial and non-commercial vineyards). Thus, the questionnaire was sent to roughly the entire population of Swiss grape growers. A total of 489 growers responded completely to the survey (response rate: 11.8 %). Questions were derived from previous sources or literature [[1], [2], [3]]. Participation was incentivised. The data was anonymised.
Data source location	Country: Switzerland Institution: ETH Zurich
Data accessibility	Repository name: ETH Zürich Research Collection [4] Data identification number: 10.3929/ethz-c-000787903 Direct URL to data: https://doi.org/10.3929/ethz-c-000787903
Related research article	None

## 1 Value of the Data

• Farm-level data collection specifically about detailed adoption of and perceptions about four key pesticide reducing measures which can substantially contribute to pesticide risk reduction in viticulture.
• Collection of detailed data on the stages of the adoption process for pesticide-reducing measures, going beyond binary adoption indicators.
• Detailed collection of labour management decisions at the farm level including future perceptions about labour issues. These include overall perspectives and those related to specific plant protection measures in viticulture.
• Researchers, policy makers, and food-value chain actors can use the data to understand barriers and determinants of the adoption of key pesticide reducing measures and their labour implications in viticulture’s tight labour environment
• The data on production choices, pest management, and labour management strategies in grape production, combined with extensive information on farmer and farm characteristics as well as behavioural traits, allow for standalone research, comparisons, and linkage with other studies or datasets.

## 2 Background

The production of grapes for winemaking is economically the most relevant crop in Switzerland. It is also the crop in which the largest quantity of pesticides is used, alone fungicides account for 27 % of all pesticides used in agriculture at large. Simultaneously, grape production is very labour intensive, with Swiss vineyard labour demand estimated at 450 to 800 h per hectare annually. Thus, the data was collected to analyse the environmental and social impacts of transitioning to low-pesticide grape growing in Switzerland, focusing on its effects on farmers and farm workers. While also eliciting production, pest and labour management decisions, it seeks to provide a basis for novel evidence generation for policy, practice as well as science on how the shift to low or no-pesticide production impacts labour demand and labour qualification.

## 3 Data Description

**Fig. 1:**
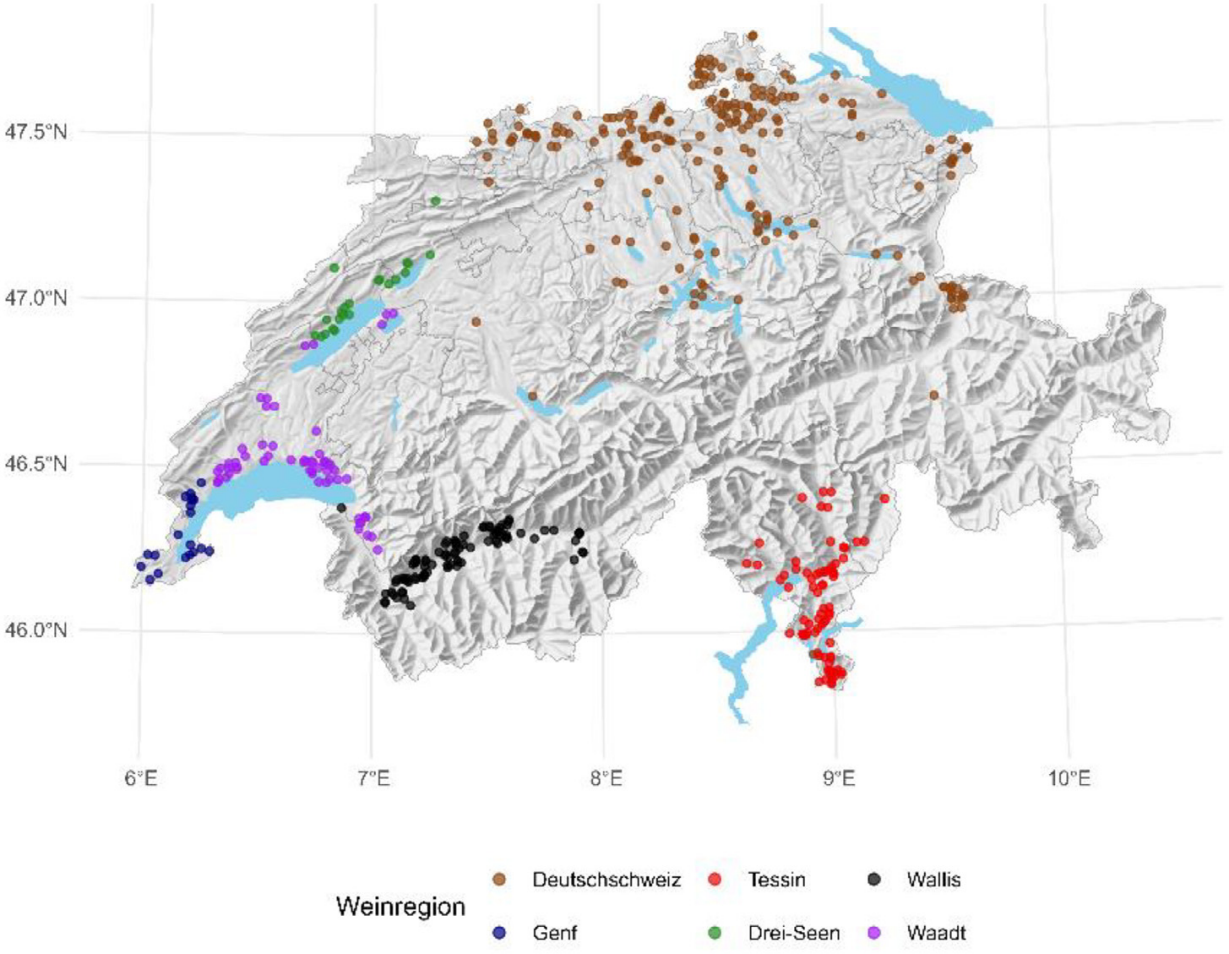
Sample overview.

*Note*: The scatters are randomly positioned within municipalities and do not represent actual locations of the farms. This is done for confidentiality reasons.

We collected survey data from 489 grape growers in Switzerland about their production, pest management and labour management decisions. More specifically, we asked growers about their current and future plantation expectations of specific varieties, their farm and farmer characteristics, their risk and time preferences and behavioural factors. We particularly focused on the adoption and perceptions of four key pesticide reducing measures, including stages of the adoption process (ranging from being unaware, aware, evaluating, trailing the measure, adoption to dis-adoption of the measure), thus going beyond binary adoption indicators. The data collection was carried out with an online survey from February 28th to April 6th, 2025, in three main official Swiss languages.^1^ Refer to Fig. 1 for an overview of the sample. The dataset, survey and codebook describing the variables are available online on the ETH Zürich Research Collection: https://doi.org/10.3929/ethz-c-000787903

In the survey, we collected extensive data on current and future cultivation of grape varieties, including fungus-resistant varieties. Data on a wide range of pest management strategies (e.g. pest control strategies against weeds, insects, and fungi) were also collected. Farm (e.g. size, production system, labelling) and farmer (e.g. age, gender, education) characteristics were collected. We elicited information on labourer numbers and their characteristics as well as grower perspectives on labour management practices. Moreover, risk preferences in four domains (production, agriculture, marketing and plant protection) and time preferences were elicited using self-assessment questions [5]. Data collected also includes information on locus of control and self-efficacy. Additionally, we focussed on four key pest management strategies and detailed adoption data as well as the grower’s perception on adoption factors and labour distortions of the strategy. The data collection builds on work by Knapp, Bravin, and Finger [2] as well as Zachmann, McCallum and Finger [1]. The collected data covers 3′207.6 hectares of land under grapes, representing 23.4 % from the total viticultural area in Switzerland (see Table 1).

**Table 1:** Sample representativeness in terms of observable characteristics.

	Sample of grape growers	Switzerland (whole farming population in 2024)	Source
**Age**	64.2 % of farms in our sample were managed by people over 50 years of age	57 % of farms in Switzerland were managed by people over 50 years of age	[6]
**Female farmers**	9.6 %	7.7 %	[7]
**Organic producers**	15.3 %	16.7 %	[7]
**Farm Size** (in ha)	8.2	22.1	[7]
**Land under grapes** (in ha)	3210 (on average 23.4 % of total farm size)	13, 690	[6]
**Share of fungus resistant varieties**	7.2 %	3.9 %	[8]

*Note*: The table summarises key characteristics of observable variables in our sample and compares their mean values with those of Swiss agriculture overall, providing representative benchmarks. For example, the share of organic producers and the proportion of growers over 50 in our sample closely align with national averages (note unofficial estimates also state that 15.3 % of Swiss vineyards are organic). However, the sample slightly overrepresents female growers and smaller farms, particularly those cultivating fungus-resistant varieties. It is important to note that vineyards and other specialty crop farms are generally smaller in farm size than other farms such as focused on arable crop production.

We integrated survey responses with meteorological data from 103 weather stations across Switzerland, matched according to the great-circle-distance to each farm. The weather data, sourced from Agrometeo [9], spans daily records from 2014 to 2024−covering ten years preceding the survey. From this, we derived an average temperature, precipitation, and infection risk indices for *Oidium* (powdery mildew) and *Peronospora viticola* (downy mildew) for the vegetative period (i.e. from April until October). For context, *Oidium* and *Peronospora viticola* are markedly the most problematic (fungal) pathogens for Swiss viticulture.

These indices assess the likelihood of infection by *Oidium* and *Peronospora viticola* using meteorological variables (temperature, precipitation, and humidity) alongside the developmental resistance of grape tissues. For instance, susceptibility varies throughout the growth cycle, with peak vulnerability occurring in June and July, and declining toward harvest (typically between late August and October, depending on grape variety, location, and year).

Note that the indices use different measurement scales: *Oidium* risk is expressed as a percentage (0 % indicating no risk, 100 % indicating high risk), while the *Peronospora viticola* index is categorical—1 for low risk, 2 for moderate risk, and 3 for high risk. To match weather station data with farm locations, we minimized the geographic distance using the haversine formula, which calculates the shortest path between two points on a sphere.

## 4 Experimental Design, Materials and Methods

We used LimeSurvey [10], an online platform, to design, implement and carry out the survey. The survey was pre-tested with 2 grape experts and a pilot study was completed with 16 grape growers in November 2024 in coordination with the centre for agricultural and viticultural education and extension of the canton of Zurich, Switzerland. As an incentive to participate in the main survey, we raffled 25 vouchers with a value of 50 Swiss Francs (CHF) each among participants who completely answered the questionnaire. Additionally, individual feedback on the survey results were provided to participants who indicated their interest. This feedback included both aggregate information and individual positions relative to peers regarding pest management choices, future expectations of changes in grape production, and perceptions of four key pesticide-reducing measures. For a reusable template of such individualised reports see https://github.com/luccalino/ParametrizedReports.

The mean time to complete the survey was 27 min and 41 s. There were 64 questions in the survey divided into the following sections:

i) Information on grape varieties
ii) Agronomic and pest management practices
iii) Farmer characteristics
iv) Farm characteristics
v) Labour management practices
vi) Behavioural characteristics
vii) Questions regarding four key pesticide reducing measures
  1. Fungus-resistant grapes
  2. Plant resistance inducers
  3. Inorganic materials
  4. Mechanical weeding

### 4.1 Grape varieties and cultivated areas

We asked survey participants about their farm size (in are, i.e. 100 square meters). Thereafter, we elicited from a multiple-choice menu of the 35 most frequently planted grape varieties in Switzerland which varieties the survey participants currently grow on their farm.^2^ Moreover, in case the participant has adopted another variety that was not listed in the menu, we provided the option to add up to 15 more varieties manually. For each variety, we asked for the area under cultivation to receive a complete picture on the variety portfolio of a farm. Furthermore, we elicited at the varietal level future plantation changes. More specifically, we asked growers which varieties they are planning on i) newly planting or ii) expanding the land under currently planted varieties or iii) explanting varieties within the next five years. Overall, we identified 140 different grape varieties used by the participants, of which 69 are fungus-resistant varieties.

### 4.2 Agronomic and pest management practices

We also asked growers about their on-farm pest management practices. Specifically, we surveyed an extensive list of employed pest management practices against weeds, insects, and fungi. These 25 practices included preventive (i.e. insect netting), biological (i.e. Pheromonal confusion), technology based (i.e. Decision support systems), and chemical-synthetic strategies (i.e. Pesticides), these practices included those used in organic and non-organic viticulture (See Table 2). Moreover, we elicited whether growers applied copper-based fungicides, the amount they applied and if they employed any mitigation strategies to reduce copper usage. We also elicited information on the phytosanitary application method used, specifically the machinery, such as hand sprayers, low drift nozzles, tunnel recycling sprayers but also drones and helicopters.

**Table 2:** Summary of pest management questions.

Question description	Possible responses
How do you impede or control for **insect and mite infestations** in your vineyard? (multiple choice)	• Confusion techniques (e.g. Pheromones) • Beneficial insects (e.g. predatory mites, beetles) • Preventive measures (e.g. Field hygiene, irrigation, plant nutrition) • Decision-making tools (e.g. Early warning systems, prognosis systems, damage threshold systems) • Chemically synthesised insecticides • Mechanical control (e.g. Nets, traps) • Non- chemical synthetic pesticides (e.g. Pyrethrin, spinosad, kaolin, oils, acids, etc.) • None • Other: __________
How do you impede or control for **weeds** in your vineyard? (multiple choice)	• Mechanical weeding (e.g. mulching, mowing, cable trimmer) • Interrow weeding • Controlled cover cropping (e.g. special seed mixtures) • Herbicides only under the vines • Herbicides on the full plot • None • Other: _____________
How do you impede or control for **fungal infections** in your vineyard? (multiple choice)	• Removal of infected material from the vineyard (e.g. Field hygiene) • Decision support tools (e.g. Early warning systems, prognosis systems, damage threshold systems) • Canopy management (e.g. thinning of clusters, air flow control, leaf removal) • Microorganisms (e.g. Bacillus subtilis, B. pumilus, Trichoderma spp., Fusarium spp) • Control of fertilizer (Potassium Phosphonates,) • Grape resistance inducers (eg. Auralis, FytoSave, Vacciplant, Fosetyl-Al) • Inorganic material (e.g. Potassium bicarbonate, Ulmasud, Myco-Sin and Myco-San) • Synthetic-chemical fungicides • Copper-containing fungicides • Sulphur- containing fungicides • None • Other: _________

### 4.3 Farmer characteristics

Farmer-specific information obtained included participants’ gender, year of birth, educational background, revenue percentages from agriculture and viticulture. We also asked what percentage of their land they were leasing and if they had a successor to their farm.

### 4.4 Farm characteristics

We asked growers for their postcode to match survey data to climate data.^3^ We asked how growers market their grapes (as grapes or as wine), and which marketing channel they use. More specifically, we elicited the percentages of produce they market as grapes to winemakers, cooperatives, or commerce, or as wine to commerce, major distributors, gastronomy or directly to consumers.

We elicited information on farm production method employed and their use of labels [2] as well as a standardised national measure describing the amount of labour employed on the farm.

We also asked participants whether the farm has taken part in one of many different agri-environmental schemes available for vineyards. Moreover, we elicited whether they received agri-environmental payments for purchases/investments of low-drift application machinery. We additionally asked them whether they were aware of an agri-environmental support scheme for fungus-resistant varieties available since 2023, and whether they have applied or plan to apply for the scheme within the next five years.^4^

Finally, we elicited sources of information, such as where they seek information for plant protection decisions and asked the growers whether they implement risk management strategies at the farm, such as through diversification strategies or formal insurance uptake.

### 4.5 Labour management practices

Since viticulture is labour-intensive, with Swiss labour demand estimated at 450 to 800 h per hectare annually [11], we asked growers about their labour management practices and their perception on labour-related difficulties. More specifically, we elicited how many annual and seasonal labourers the farm employs, and how long seasonal labourers are employed for on average. Thereafter, we asked the growers about who they hire as employees (e.g. apprentice, EU (European Union) labourers, non-EU labourers, Swiss citizens) and how they hire employees (e.g. through personal contacts, contractors or agencies). These questions were asked separately for annual and seasonal employees.

Additionally, we asked an array of labour related questions, such as their perception on labour acquisition difficulties, if these difficulties impact vineyard decisions, and whether they see mechanisation as a solution to mitigate these difficulties. Furthermore, we elicited whether growers expect labour acquisition to become more difficult in the next years. Finally, we asked which group of labourers apply plant protection measures.

### 4.6 Behavioural characteristic

We elicited time and risk preferences that are expected to partially explain decisions made by growers [12]. For time preferences, we followed Falk et al. [5] and asked growers how willing they are to give up income that is beneficial for them or their farm today in order to benefit more from that in the future. We used a 11-point Likert scale, ranging from not willing (0) to very willing (10) to give up income. For risk preferences, growers were asked on an analogous 11-point Likert scale how willing they were to take or mitigate risks in the areas of production, market and prices, plant protection, and agriculture in general, respectively [2].

We also added questions on locus of control [13] and self-efficacy [14] to our questionnaire as these concepts are relevant to explain growers’ pest management decisions [15]. We phrased our questions regarding self-efficacy similar to Knapp et al. [2] and those for locus of control based on Abay et al. [16]. Therefore, locus of control and self-efficacy were measured with 5-points Likert scales and related to grape/wine production. We included seven questions overall, three on locus of control and four questions on self-efficacy with answer options ranging from strongly disagree to strongly agree.

### 4.7 Elicitation of adoption stages of key pesticide reducing measures

In the last part of the survey, we elicited the stages of adoption of four key pesticide reducing measures in viticulture. These measures are part of the 25 aforementioned general measures (see Table 2). These key measures were chosen due to their high pesticide reduction potential and middling uptake in Swiss viticulture. More specifically, we asked growers for each pesticide reducing measure (i.e. the plantation of fungus-resistant varieties, the use of plant resistance inducers, inorganic materials and mechanical weeding) which is the furthest adoption stage that describes the current use of the measure best. We included answer options following the adoption stages described in Weersink and Fulton [3], namely (un)awareness of the measure, evaluating or trialling the measure and adoption or dis-adoption of the measure. Those trialling or adopting are then asked the percentage of their land that they apply this measure, and if they seek to increase or decrease this percentage.

Regardless of their adoption stage, we then asked two array questions about their perceptions or experiences of socioeconomic impacts and adoption factors specific to the use of the pesticide reducing measures. These questions focused on the measures’ impact on labour demand, fixed and variable costs, production risk, labour skill requirement and labour acquisition difficulties. For the exact survey questions See Table 3.

**Table 3:** Summary of Socioeconomic impacts of four key pesticide reducing measures.

Description	Survey Question
Labour Demand	How does use of [measure] affect labour demand?
Fixed costs	How do fixed costs change with the use of [measure] (in comparison to not using them)?
Variable costs	What are the variable costs of using [measure] (compared to not using them)? (e.g. application costs, etc.)
Production risk	Are there changes in production risk associated with the use of [measure]?
Skill requirement	How does the use of [measure] change the skill requirements of your workforce?
Labour acquisition	How does the use of [measure] affect the difficulty of procuring labour?

Note: questions in Table 3 were focused on the following pesticide reducing measures: the plantation of fungus-resistant varieties, the use of plant resistance inducers, inorganic materials and mechanical weeding.

For following adoption factors the grower was asked how they impact their propensity to adopt or utilise the pesticide reducing measure. The adoption factors elicited are knowledge and economic, environmental, health, social, and external factors. This grouping follows the literature on adoption factors. More specifically, knowledge on certain practices or information sources such as the presence of local agricultural authorities, and some informative policy regulations have been shown to motivate higher adoption rates of sustainable farming practices [17]. Economic viability is a key driver of adoption and innovation by growers [18]. Due to the list of negative externalities from pesticide usage on environmental and human health, the consciousness of these health risks has been observed to incite adoption of sustainable viticultural practices [19]. Social factors such as social consciousness in residential areas and spillover effects have been known to drive adoption of no or low pesticide practices [20]. Additionally, pest pressure varies within the microclimates of individual plots, thus these external factors can dictate pest management decisions. Both questions elicit responses on a Likert scale from 1 (stark negative impact) to 5 (stark positive impact).

## Limitations

Not applicable

## Ethics Statement

The survey has been approved by the Ethics Commission of ETH Zurich on November 26, 2024, with confirmation number 24 ETHICS-385. All participants declared informed consent prior to beginning the survey.

## CRediT Author Statement

**Philipp Höper**: Conceptualisation, Methodology, Writing – original draft, Data Preparation, Data Analysis. **Lucca Zachmann**: Conceptualisation, Methodology, Writing – Reviewing & Editing, Visualisation, Data Preparation, Data Analysis. **Robert Finger**: Conceptualisation, Methodology, Writing – Reviewing & Editing, Funding acquisition.

## Data Availability

ETH Research CollectionData on Swiss grapevine growers’ production, pest and labour management decisions (Original data).

## Appendix Supplementary materials

Image, application 1

## Footnotes

1 We contacted the growers either in German, French or Italian based on the main language spoken in their municipality. Growers were then able to select their preferred language for the survey.

2 We asked participants to only mention the varieties with a cultivated on an area over or equal to 1 are.

3 Note that we do not provide growers’ postcode in the openly accessible dataset for confidentiality reasons.

4 For an overview of agri-environmental programs visit the website of the Federal Office of Agriculture at https://www.blw.admin.ch.
