# Supplementary material for: Data on Swiss grape growers’ production, pest and labour management decisions
Source: Data Brief. 2025 Dec 24;64:112421. doi: 10.1016/j.dib.2025.112421 (PMC12813452; doi:10.1016/j.dib.2025.112421)
Supplement: Supplementary file 1 [file mmc1.docx]

Supplementary material – Data Description table

| **Question/ Description** | **Answer options** | **Coding** |
| --- | --- | --- |
| Wine region in which the participants' farm is located | - | Deutschschweiz, Ticino, Vaud, Geneva, Trois lacs, Valais |
| Canton in which the participants’ farm is located | - | Based of Cantonal Abbreviation |
| The language chosen to fill out the survey | - | de: German; fr: French; it: Italian |
| What is your gender? | The gender of the farmer | M: male, F: Female |
| In which year were you born? | The age of the farmer | Age [in years] |
| What is your total farmland in are (1 Are = 10m x 10m)? | The size of the farm [in are] |  |
| Please indicate the cultivated area per [VARIETY] in ares below. | Current land under [VARIETY NAME] [in are] | Numeric or NA if not indicated |
| Which of your currently grown varieties are you expecting to reduce the land within the next 5 years? | Land reductions of currently planted [VARIETY NAME] in the next five years [in are] | Numeric or NA if not indicated |
| Please indicate the land planned under the newly planted [VARIETY] in ares below. | New planting of [VARIETY NAME] in the next five years [in are] | Numeric or NA if not indicated |
| Please indicate the expanded area planned per [VARIETY] in ares below. | Expansion of currently planted [VARIETY NAME] in the next five years [in are] | Numeric or NA if not indicated |
| Are you expecting to newly plant or increase the land under some planted varieties in the next 5 years? | Yes, I am expecting to newly plant some varieties in the next 5 years. | Binary: Yes: 1; No: 0 |
|  | Yes, I am expecting to increase the land under some planted varieties in the next 5 years | Binary: Yes: 1; No: 0 |
|  | No | Binary: Yes: 1; No: 0 |
|  | I don't know | Binary: Yes: 1; No: 0 |
| Are you expecting to explant or reduce the acreage of some planted varieties in the next 5 years? | Yes | Binary: Yes: 1; No: 0 |
|  | No | Binary: Yes: 1; No: 0 |
|  | I don’t know | Binary: Yes: 1; No: 0 |
| Which factors do you consider to currently have the biggest negative impact on your grapevine yield (quality and quantity)? | Hail damage | Binary: Yes: 1; No: 0; Other: String |
|  | Fungal Infections |  |
|  | Insects infestations or pest infestations |  |
|  | Droughts |  |
|  | Weeds |  |
|  | Frost |  |
|  | Other |  |
| How do you control for insect and mite infestations in your vineyard? | Confusion techniques (e.g. Pheromones) | Binary: Yes: 1; No: 0; Other: String |
|  | Promotion of beneficial insects (e.g. predatory mites, beetles) |  |
|  | Preventive measures (e.g. Field hygiene, irrigation, plant nutrition) |  |
|  | Decision support tools (e.g. Early warning systems, prognosis systems, damage threshold systems) |  |
|  | Chemically synthesised insecticides |  |
|  | Mechanical control (e.g. Nets, traps) |  |
|  | Non-chemical synthethic plant protection products (e.g. Pyrethrin, spinosad, kaolin, oils, acids, etc.) |  |
|  | Farmer doesn't control for insect infestations |  |
|  | Other |  |
| How do you impede or control for weeds in your vineyard? | Mechanical weeding ( e.g. mulching, growing, cable trimmer) | Binary: Yes: 1; No: 0; Other: String |
|  | Interrow weeding |  |
|  | Controlled cover cropping (e.g. special seed mixtures) |  |
|  | Herbicides only under the vines |  |
|  | Herbicides on the full plot |  |
|  | Farmer doesn't control for weeds |  |
|  | Other |  |
| How do you control for fungal infections in your vineyard? | Remove infected material from the vineyard (e.g. Field hygiene) | Binary: Yes: 1; No: 0; Other: String |
|  | Decision support tools (e.g. Early warning systems, prognosis systems, damage threshold systems) |  |
|  | Canopy management (e.g. thinning of clusters, air flow control, leaf removal) |  |
|  | Use of microorganisms (e.g. Bacillus subtilis, B. pumilus, Trichoderma spp., Fusarium spp) |  |
|  | Control of fertilizer |  |
|  | Grapevine resistance inducers(eg. Auralis, fytoSave, Vacciplant, Fosetyl-Al) |  |
|  | The use of inorganic materials (e.g. Potassium bicarbonate, Ulmasud, Myco-Sin and Myco-San) |  |
|  | Synthetic-Chemical fungicides |  |
|  | Copper-containing fungicides |  |
|  | Sulphur- containing fungicides |  |
|  | Farm doesn't control fungal infections |  |
|  | Other |  |
| On average, how much copper do you apply on your grapevines (in kilograms, per hectare per year)? | Average farm-lelvel copper quantity (active substance) which farm used per ha/year in kg | Numeric |
| Do you implement measures to reduce the amount of copper applied on your grapevines? | Whether the farm implemented measures to reduce the amount of copper | Binary: Yes: 1; No: 0 |
| What copper reducing measures do you apply? | Preventive measures (e.g. pruning to reduce leaf moisture, balance fertilization to avoid excessive growth, field hygiene, canopy management, rain shelters | Binary: Yes: 1; No: 0; Other: String |
|  | Specific precision application machines (e.g. drones) or equipment (e.g. low drift nozzles) |  |
|  | Disease forecasting model (e.g. Agrometeo) |  |
|  | Variable copper dosages in relation to the disease pressure or copper fungicides with improved formulations |  |
|  | Copper alternatives of natural origin( non-chemical synthetic) (e.g. calcium carbonate, calcium hydroxide, plant extract, microbial biocontrol agents or derivates, plant extracts) |  |
|  | Application of more synthetic chemical fungicides |  |
|  | Resistance inducers (e.g. cos-oga) |  |
|  | Plant fungus-resistant varieties |  |
|  | Pathogen resilient trellis and raining systems |  |
|  | Planting less susceptible traditional Vitis varieties |  |
|  | Other |  |
| How are plant protection products applied on vines? | Hand sprayer | Binary: Yes: 1; No: 0; Other: String |
|  | Tractor |  |
|  | Closed-cabin tractor |  |
|  | Low drift nozzle |  |
|  | Spraying equipment with horizontal air Assistance |  |
|  | Gun |  |
|  | Tunnel recycling sprayer |  |
|  | Helicopte/drones |  |
|  | Other |  |
| Do you have formal training in agriculture/viticulture from any of the following? | EFZ (e.g. Farmer, Winzer, Weintechnologe) | Binary: Yes: 1; No: 0; Other: String |
|  | Agricultural management school/ "Meister" degree |  |
|  | Higher technical school (HF) |  |
|  | University of applied science (FH) |  |
|  | University or ETH |  |
|  | Fachbewilligungskurs Pflanzenschutz |  |
|  | Further education in plant protection |  |
|  | None of the above |  |
|  | Other |  |
| Which percentage of your earning are from farming? | - | A1: "0%"; A2: "1-25%"; A3: "26-50%"; A4: "51-75%"; A5: "76-100%" |
| How much is viticulture contributing to your farming income (in percent)? | - | A1: "0%"; A2: "1-25%"; A3: "26-50%"; A4: "51-75%"; A5: "76-100%" |
| What share of your farmland are you leasing? | The leased share of the farmland | A1:"0-25%", A2: "26-50%", A3:" 51-75%", A4: "76-100%" |
| Do you have a successor to your farm? | Does the farmer has a successor to their Farm | A1: "Yes"; A2: "No"; A3: "Not yet relevant" |
| How are you marketing your grapevines? | Grapes | Binary: Yes: 1; No: 0 |
|  | Wine |  |
| How are you marketing your wines (in percent)? | Direct marketing | Numeric (0-100) |
|  | Sales to commerce |  |
|  | Sales to major distributors |  |
|  | Sales to gastronomy |  |
| Do you sell your wine under any of the following labels or terms? | Bio-Suisse label | Binary: Yes: 1; No: 0; Other: String |
|  | Demeter |  |
|  | Vinatura |  |
|  | Organic- without certification |  |
|  | PIWI |  |
|  | IP Suisse |  |
|  | Fair & Green |  |
|  | None |  |
|  | Other |  |
| According to which production system do you manage your farm? | OELN/VITISWISS | Binary: Yes: 1; No: 0; Other: String |
|  | Organic (uncertified) |  |
|  | Organic (certified) |  |
|  | Integrated production ( IP Suisse) |  |
|  | Bio-dynamic |  |
|  | Vario |  |
|  | None |  |
|  | The farmer manages their farm according to something different |  |
| How many units of standard manpower does your farm have? | The units of standard manpower |  |
| What direct payment programmes does the farm participate in for vines? | Slope contribution vineyards 30-50% | Binary: Yes: 1; No: 0 |
|  | Slope contribution vineyards 50% |  |
|  | Terraces (slope>30%) (for vines) |  |
|  | Vineyards with natural biodiversity (QII) |  |
|  | Adequate cover of the soil (for vines) |  |
|  | Networking contribution for biodiversity promotion and trees (for vines) |  |
|  | Contribution for organic farming for speciality crops (for vines) |  |
|  | Contribution for the cultivation of areas with permanent crops with aids according to organic farming (for vines) |  |
|  | Contribution for the renunciation of insecticides, acarides and fungicides after flowering in permanent crops ( outside the use of plant protection products according to organic regulations] (for vines) |  |
|  | Contribution to the avoidance of herbicides in specialised crops (for vines) |  |
|  | Contribution to functional biodiversity Beneficial insect strips (for vines) |  |
|  | none |  |
| Are you aware of the structural improvement payments dedicated to fungus-resistant grapes? | - | Binary: Yes: 1; No: 0 |
| Have you or will you file a request for structural improvement payments for fungus-resistant grapes in the next 5 years? | Yes. I have filed a request | Binary: Yes: 1; No: 0 |
|  | Yes, I will file a request (within the next five years) |  |
|  | No, I will not file a request within the next 5 years |  |
| Have you received direct payments for the following purchases/investments of application methods? | 25% acquisition cost for spray blower with horizontal air flow control (max 6000.-CHF) | Binary: Yes: 1; No: 0 |
|  | 25% acquisition cost for spray blower with horizontal air flow control and vegetation detector (max 10 000.- CHF) |  |
|  | None of the above |  |
| Where do you search for your plant protection information? | Via internet | Binary: Yes: 1; No: 0; Other: String |
|  | Cantonal information services (e.g. Kantonale fachstellen, Kompetenzzentren) |  |
|  | Plant protection firms/ suppliers |  |
|  | Social media (e.g. Facebook, Twitter (X), Youtube ect. |  |
|  | Specialist articles / journals |  |
|  | Agroscope |  |
|  | FiBL |  |
|  | Other farmers/colleagues |  |
|  | The farmer did not search for new information |  |
|  | Other |  |
| What do you do to protect yourself against financial risks? | Hail insurance | Binary: Yes: 1; No: 0; Other: String |
|  | Hail and frost insurance |  |
|  | Off-farm diversification (e.g. other companies, real estate) |  |
|  | Store wine in the cellar |  |
|  | Off-farm work (e.g. consulting, tourism) |  |
|  | Agriculture-related diversification on farm ( e.g. agrotourism) |  |
|  | Creation of financial reserves (saving for bad times) |  |
|  | Forestry work |  |
|  | Direct marketing |  |
|  | Other |  |
| How many of the following type of labourers do you employ (including yourself)? -annual labourers | Number of annual labourers  (permanent full-time employees in the last 12 months) | Numeric |
| How many of the following type of labourers do you employ (including yourself)?-seasonal labourers | Number of seasonal labourers  (temporary employees who help out, e.g. during the harvest and/or all other seasonal activities) | Numeric |
| How long are seasonal workers employed on average every year (in weeks)? | Average duration of seasonal labourer employment | Numeric |
| How and who do you hire (as) annual laboures? | Through personal contacts | Binary: Yes: 1; No: 0; Other: String |
|  | Through external contractors or agencies (e.g. agrimpulse,Terremploi) |  |
|  | Swiss citizens or Permanent residents of Switzerland |  |
|  | EU labourers |  |
|  | Non-EU international labourers |  |
|  | Employing previous apprentices |  |
|  | Providing apprenticeships |  |
|  | Other |  |
| How and who do you hire (as) seasonal labourers? | Through personal contacts | Binary: Yes: 1; No: 0; Other: String |
|  | Through external contractors or agencies (e.g. agrimpulse,Terremploi) |  |
|  | Swiss citizens or Permanent residents of Switzerland |  |
|  | EU labourers |  |
|  | Non-EU international labourers |  |
|  | Other |  |
| To what extent do you agree or disagree with the following statements about your experience of labour? | I experience difficulties finding the necessary number of Annual labourers to fill vacancies | A1: "Strongly disagree": A2: "Disagree"; A3: "Neither agree or disagree"; A4: "Agree"; A5: "Strongly agree" |
|  | I experience difficulties finding the necessary number of Seasonal labourers to fill vacancies |  |
|  | My vineyard decision making ( such as vineyard expansion, variety choice, or structural changes) is affected by the difficulties to reach labour demand |  |
|  | I see increased mechanisation and automation in my vineyard as a solution to insufficient labour |  |
|  | I expect the difficulty of the acquisition of Seasonal labourers to increase in the next 5 years |  |
| Who applies plant protection products on your farm? | Annual labourers | Binary: Yes: 1; No: 0; Other: String |
|  | Seasonal labourers |  |
|  | Household labour |  |
|  | Farmer himself |  |
|  | External labourers/ contactors (e.g. drone pilots) |  |
|  | other |  |
| How willing are you to give up income that is beneficial for you/the farm today in order to benefit more from that in the future? | - | from 0 (= Not willing) to 10 (= Very willing) |
| Are you willing to take risks or do you try to mitigate risks in the areas mentioned below? | Production | from 0 (= Not willing) to 10 (= Very willing) |
|  | Market and prices |  |
|  | Plant protection |  |
|  | Agriculture in general |  |
| Do you agree or disagree with the following statements about your attitudes towards grape production? | When I encounter difficulties in grape/wine production, I can usually think of a solution | 1: "Strongly disagree": 2: "Disagree"; 3: "Neither agree or disagree"; 4: "Agree"; 5: "Strongly agree" |
|  | I am confident that I can accomplish my production goals at the end of the harvest |  |
|  | I can solve production issues if I invest the necessary effort |  |
|  | How successful my grape/wine production depends mostly on my skills as a farmer |  |
|  | Grapevine growing is more dependent on the weather than on what I do |  |
|  | Success in grapevine production can only be slightly influenced by farmers |  |
|  | I usually set myself quite ambitious production goals |  |
| Station | Nearest weather station to the farm | String |
| Average *peronospora* *viticola* infection risk index between 2012 and 2024 from the nearest station | - | 1: low risk; 2: moderate risk; 3: high risk |
| Average *oidium* infection risk index between 2012 and 2024 from the nearest station | - | 0 (no infection risk) to 100% (high infection risk) |
| Average temperature between 2012 and 2024 form the nearest station | - | in degrees celcius (°C) |
| Average precipitation between 2012 and 2024 from the nearest station | - | in milimeters (mm) |
| **The following Questions are asked four times; once for each measure (planting fungus-resistant grapevines, the use of plant resistance inducers, inorganic materials and mechanical weeding)** | | |
| Question | Description | Coding |
| Which stage best describes your current use of [the measure]? | The current stage of the adoption of [the measure] | A1:"Unknown", A2:"known", A3:"Pre-Trial Evaluation", A4:"Trialing", A5:"Adopted", A6: "Dis-Adopted" |
| On what percentage of your land do you use [the measure]? | The percentage of the farmland where [the measure] is used | A1:"0-25%", A2: "26-50%", A3: "51-75%", A4: "76-100%" |
| Do you see any changes in your usage of [the measure] in the next 5 years? | Will the farmer increase/maintain or decrease the usage of [the measure] in the next 5 years | A1:"Increase", A2: "maintain", A3:"decrease" |
| How does the adoption of [the measure] effect labour demand? | Change in labour demand because of adoption of [the measure] | A1:"Stark decrease, A2:" decrease", A3: "No change", A4: "increase", A5: "Stark increase" |
| How do fixed costs change with the adoption [measure] (in comparison to non-adoption)? (e.g. costs for new machines, systems, etc.)? | Change in fixed costs because of adoption of [the measure] | A1:"Stark decrease, A2:" decrease", A3: "No change", A4: "increase", A5: "Stark increase" |
| How do variable costs change with the adoption [measure] (in comparison to non-adoption)? (e.g. application costs, ect.)? | Change in variable costs because of adoption of [the measure] | A1:"Stark decrease, A2:" decrease", A3: "No change", A4: "increase", A5: "Stark increase" |
| Are there changes in production risk related to adopting [the measure]? | Change in production risks because of adoption of [the measure] | A1:"Stark decrease, A2:" decrease", A3: "No change", A4: "increase", A5: "Stark increase" |
| How does the usage of [the measure] change the skill requirements of your workforce? | Change in skill requirement because of [the measure] | A1:"Stark decrease, A2:" decrease", A3: "No change", A4: "increase", A5: "Stark increase" |
| How does the introduction of [the measure] affect the difficulty of procuring labour? | Change in difficulty of labour acquisition because of introduction of [the measure] | A1:"Stark decrease, A2:" decrease", A3: "No change", A4: "increase", A5: "Stark increase" |
| How do the following factors impact your propensity to adopt [the measure]? | Impact of Knowledge on propensity to adopt [the measure]? (e.g. available information/knowledge about [the measure] or their long-term effects i.e. resistance decay) | A1:"Stark negative effect", A2:" negative effect", A3: "No effect", A4: "positive effect", A5:" stark positive effect" |
|  | Impact of Economic Factors on propensity to adopt [the measure] (e.g. marketability, access to Direct Payment programs or private mark-up schemes) |  |
|  | Impact of Environmental Factors on propensity to adopt [the measure] (e.g. the impact of [the measure] on the environment and soil health) |  |
|  | Impact of Human Health on propensity to adopt [the measure] (e.g. the impact of the use of [the measure] on the health of Labourers and Neighbours) |  |
|  | Impact of Social factors on propensity to plant fungus-resistant varieties (e.g. the use of [the measure] by fellow winemakers, the social acceptance of the measure, the social credibility gained using [the measure]) |  |
|  | Impact of External factors on propensity to adopt [the measure] (e.g. [the measure] is suitable for specific parcels of land) depending on slope, aspect, moisture and microclimate) |  |
